# Physician presence at out-of-hospital cardiac arrest is not necessarily the cause of improved survival

**DOI:** 10.1186/s13049-016-0282-8

**Published:** 2016-07-04

**Authors:** Pieter Francsois Fouche, Paul Andrew Jennings

**Affiliations:** Department of Community Emergency Health and Paramedic Practice, Monash University, Building H McMahons Road, Frankston, Melbourne, 3199 Australia

**Keywords:** Cardiac arrest, Out-of-hospital, Confounding

## Abstract

A recent publication Hiltunen et al. on Out-of-Hospital Cardiac Arrest (OHCA) in Finland show increased survival when a physician attends an OHCA, compared to EMS. But it is likely that physicians attend OHCA patients with a different prognosis due to comorbidity or illness severity, which causes confounding by indication and is the likely cause for the physician and survival association.

## Letter

Dear Editor

It is with interest that we read the paper by Hiltunen et al. on Out-of-Hospital Cardiac Arrest (OHCA) in Finland [1]. They found that the presence of a physician at an OHCA is significantly associated with improved survival. This finding would have important implications for the clinical skill mix of OHCA teams. But Hiltunen and colleagues rightly advise that their finding of physician associated survival should be interpreted cautiously. Hiltunen et al. state that it is not uncommon for third tier (physician) EMS to attend OHCA *only after they were tasked* to that OHCA upon the information from first and second tier EMS. This means that physician based EMS in Finland might selectively treat OHCA where the patient lived long enough for the physician EMS to attend, which might mean that OHCA treated by physicians have a better prognosis. Furthermore, it is possible that first and second tier EMS personnel request the attendance of physicians at cases which are deemed potentially viable rather than those with characteristics known to be associated with poorer outcomes. This phenomenon is called confounding by indication and happens when patients that are “sicker” receive an intervention preferentially [2]. Confounding caused by sicker patients is a problem that besets cardiac arrest research and causes the results of such research to be biased [3, 4].

We believe that the maldistribution of prognosis between the physician and non-physician EMS caused confounding by indication and that this confounding could explain the results of the Hiltunen study. Hiltunen et al. mention that the improved survival associated with physician presence is in disagreement with a previous OHCA study from Norway [5]. However, their finding of physician benefit might be real and should be further investigated. It is important that such a study should adjust for confounding caused by sicker patients using illness severity scores and comorbidity indices such as the Pittsburgh Cardiac Arrest Category illness severity score and Charlson index [3]. If a positive association of physician- attended OHCA and survival remains it will be an important finding.

## Abbreviation

OHCA, Out-of-hospital Cardiac arrest

## References

[1] Hiltunen P, Jantti H, Silfvast T, Kuisma M, Kurola J (2016). Airway management in out-of-hospital cardiac arrest in Finland: current practices and outcomes. Scand J Trauma Resusc Emerg Med.

[2] Porta M (1997). Confounding by indication and past clinical trials. Epidemiology.

[3] Fouche PF, Carlson JN (2016). The importance of comorbidity and illness severity scores in cardiac arrest research. Resuscitation.

[4] Fouche PF (2015). Out-of-hospital cardiac arrest studies must adjust for sicker patients properly. Am J Emerg Med.

[5] Olasveengen TM, Lund-Kordahl I, Steen PA, Sunde K (2009). Out-of hospital advanced life support with or without a physician: effects on quality of CPR and outcome. Resuscitation.

